# 3D Face Point Cloud Reconstruction and Recognition Using Depth Sensor

**DOI:** 10.3390/s21082587

**Published:** 2021-04-07

**Authors:** Cheng-Wei Wang, Chao-Chung Peng

**Affiliations:** Department of Aeronautics and Astronautics, National Cheng Kung University, Tainan 701, Taiwan; f04066010@gs.ncku.edu.tw

**Keywords:** point cloud, 3D face reconstruction, 3D face recognition, iterative closest point (ICP), principal component analysis (PCA), k-means, DBSCAN

## Abstract

Facial recognition has attracted more and more attention since the rapid growth of artificial intelligence (AI) techniques in recent years. However, most of the related works about facial reconstruction and recognition are mainly based on big data collection and image deep learning related algorithms. The data driven based AI approaches inevitably increase the computational complexity of CPU and usually highly count on GPU capacity. One of the typical issues of RGB-based facial recognition is its applicability in low light or dark environments. To solve this problem, this paper presents an effective procedure for facial reconstruction as well as facial recognition via using a depth sensor. For each testing candidate, the depth camera acquires a multi-view of its 3D point clouds. The point cloud sets are stitched for 3D model reconstruction by using the iterative closest point (ICP). Then, a segmentation procedure is designed to separate the model set into a body part and head part. Based on the segmented 3D face point clouds, certain facial features are then extracted for recognition scoring. Taking a single shot from the depth sensor, the point cloud data is going to register with other 3D face models to determine which is the best candidate the data belongs to. By using the proposed feature-based 3D facial similarity score algorithm, which composes of normal, curvature, and registration similarities between different point clouds, the person can be labeled correctly even in a dark environment. The proposed method is suitable for smart devices such as smart phones and smart pads with tiny depth camera equipped. Experiments with real-world data show that the proposed method is able to reconstruct denser models and achieve point cloud-based 3D face recognition.

## 1. Introduction

In the past, the technology and algorithm of hardware has not been well developed. Related work about facial reconstruction and recognition are mainly based on 2D image processing. In recent years, developments in neural networks and statistics have greatly influenced the field of computer vision. Benefitting from mature techniques and lower costs of computers, fast computing speed is no longer leaving researchers in the dust. Therefore, the applications of 3D point cloud are broadly used in object detection [1,2,3], reconstruction [4,5] and recognition [6,7].

As the rapid development of artificial intelligence (AI) and the internet of things (IoT), personal privacy is getting a lot of discussion. In recent years, researchers have studied intensively on protecting personal data facilitatively. Furthermore, the most relevant issue is facial analysis, which can be applied to facial recognition for high security systems [8,9,10], health care [11,12], law enforcement [13,14], etc. Recently, 3D face reconstruction has been a major trend and challenging work in computer vision field, which provides comprehensive face geometry and invariant to illumination compared with 2D imaging. However, previous works [15,16,17,18] show that 3D face reconstruction approaches are mainly based on 2D images, for example, shape from motion (SFM) [19], shape from shading (SFS) [20], multi-view geometry [18], etc. In addition, these strategies may require complicated instrument establishing, which is time consuming, and the accuracy of reconstruction models are often affected by illumination variations. S. Zhang et al. [21] proposed a method that reconstructed dense 3D face point clouds by applying a radial basis function (RBF) with a single depth image of Kinect data. The first stage aligned the depth data with RGB image. Then, detecting facial region by using the Haar Cascade Classifier [22], the raw face point cloud can be obtained. To acquire more accurate face point cloud, the second stage applied K-means clustering [23] to divide the clusters into facial regions and others. After that, the authors employed k Dimensional-tree (kD-tree) to find the neighborship and distance between points to perform interpolation based on RBF. The result [21] shows that the number of points is ten times denser than the initial point cloud. However, the accuracy might be affected by facial orientation when performing facial recognition because only a single frame is taken. In this paper, we present an algorithm that reconstructs the whole head point cloud from multi-view geometry. Not only are the ears preserved, but insignificant parts like shoulders are removed. The procedures are done without any RGB information, which verify the feasibility of 3D face reconstruction/recognition in a low-light environment or a fully dark situation.

A 3D point cloud is composed of several points in a three-dimensional space which represents the shape and surface of an object. Moreover, features such as curvature and normal are also included. Point clouds can be acquired directly from a stereo camera, laser scanner or light detection and ranging (LiDAR). Nowadays, the research and related algorithm of 3D point cloud processing are trending issues, since data acquisition is no longer difficult. As autonomous cars are increasingly driving on the road, safety is the most concerned topic for drivers. Building a reliable vehicle to satisfy customers, multiple sensors such as LiDAR and cameras are necessary to be equipped and several modules based on these devices must be integrated. The related sensing algorithms such as object detection and environment perception are bound up with 3D point clouds. In regard to [24], the authors reviewed the techniques used in autonomous car for point cloud processing, including the overview of map creation, localization and perception modules. To integrate these modules for security, 3D point cloud learning such as reconstruction, classification and segmentation are elementary tasks to deal with.

In the study of point cloud pre-processing techniques, feature extraction is the most significant process which help us to detect certain types of points with different information. Fast point feature histogram (FPFH), which is a descriptor that can represent features of local area, was proposed by Rusu, et al. [25]. Given a point cloud, then searching k-nearest neighbors (kNN) in the sphere with a selected radius for each point. The surface normal can be estimated between the point pairs. Point feature histogram (PFH) at a query point is related to its neighbors and the normal of surrounding points. Considering the angular variation of point pairs, local features can be described by using PFH. However, this approach may be very time-consuming.

While performing facial feature extraction, normal and curvature will be the essential concepts to analyze a 3D point cloud set. Principal component analysis (PCA) is the most commonly used method and is simple to realize [26]. The dimensionality may be quite high when dealing with a massive dataset. PCA is also a dimension-reducing approach that simplifies the data by reducing the number of less important variables. Moreover, information of the original dataset can still be hold by its covariance matrix. The concept of PCA can not only be used in 2D images, but it also be extended to extract 3D point cloud features by computing the eigenvalues and eigenvectors of the covariance matrix [27]. In this paper, the PCA is applied for extracting features from a face point cloud to measure similarity and detect the position of important parts of the face, e.g., nose tip and eyes, which possess higher variations of curvature.

As mentioned above, the applications of 3D face recognition have developed rapidly in our life. The advantages of face recognition with an additional dimension can be summarized [28]: (a) invariant to illumination, pose, viewpoint; and (b) potential for higher recognition accuracy because the features such as normal and curvature on faces are better described rather than a 2D image. According to the type of facial point cloud matching strategies, there are several categories.

Spatial geometry approach: Nunes et al. [29] performed face recognition by using a face curvature map (FCM) and experimenting with the VAP RGB-D Face Database. Projecting the 3D point cloud data on 2D image and representing features such as normal and curvature index with different colors. The similarity determination is done by FCM histogram matching. Medioni et al. [30] acquired 3D facial shapes from passive stereo sensor that recovered depth information with mesh of triangles. Then, they performed face recognition using ICP to match the face surface. However, the system set-up needs a large working envelope and requires two calibrated cameras, which is too complicated to implement.

Deep learning approach: Jhuang et al. [31] experimented with Kinect and estimated normal and curvature of face point cloud by using the Point Cloud Library (PCL). The PCL is an open-source library for point cloud processing, including filtering, registration, model fitting, object segmentation, etc. After that, a deep belief network (DBN) with 800 training epochs is adopted to train these extracted features. Finally, the pre-trained model is used to identify whether the input face point cloud feature is same person. The experiment proved that the accuracy might decrease with the increment of hidden layers. However, this approach poses a heavy loading of computation and a huge training dataset is needed.

Local features approach: Lee et al. [32] performed face recognition using curvature at eight feature points on face. Calculating the relationship of distances and angles between these points, that is, relative features. In the recognition stage, a feature-based support vector machine (SVM) is applied for classification. However, the paper didn’t mention how the feature points are detected. In other words, the points were probably located manually.

In this paper, we present a complete procedure from the very front data acquisition to the face point cloud reconstruction, and finally apply the untrained 3D point cloud data to facial recognition. Briefly, a robust stitching method for multi-view face point cloud reconstruction is applied to rebuild a much denser 3D face point cloud with high-density, numerous-feature points. The reconstruction models are taken as reference models. Next, certain feature extractions, as well as a similarity score, are used to recognize whether the input 3D point cloud is the most similar as one of the models in database. The algorithm presented in this paper is to verify the feasibility of 3D face reconstruction and recognition, which is applied for some specific fields such as our laboratory and office. Hence, we collect our own data set with total 1412 frames in the testing phase. The result shows that the algorithm is competitive with a processing time of less than 0.23 s and a high accuracy of almost 100%. Besides, all algorithms are implemented with low-cost devices. The CPU of the device is i7-6700HQ. Any GPU capacity and complicated deep learning algorithm was not applied. The main contributions of this work include: (a) ROI confinement techniques for precise head segmentation by K-means clustering; (b) computing efficiently and alignment skills for multi-view point clouds registration by ICP; (c) simple method to detect outliers by DBSCAN and face feature extraction; and (d) a novel face registration similarity score is presented to evaluate 3D face point cloud recognition.

## 2. 3D Point Cloud Preprocessing

To self-contain this work, in this section, numerous fundamental algorithms will be illustrated briefly. The well-known Microsoft Kinect v2 is employed to acquire the body point cloud from different views. The multi-view 3D point clouds are going to rebuild a high-density model. Point cloud registration using ICP plays an important role in reducing the deviation of distance between point clouds. In addition, approaches for feature extraction, segmentation and denoising will also be introduced.

### 2.1. Principal Component Analysis (PCA)

As previously mentioned, feature extraction is the significant process when dealing with massive data. PCA [26,27] is a dimension-reducing approach that simplifies the data by reducing the number of variables. The number of features in data is regarded as the dimensionality of point set. Extracting important components from the features, that is, reducing the dimensionality which make the composition of data not too complex and convenient to analyze.

The main concept of PCA is to find a vector in feature space that the largest variance of data can be acquired through orthogonal projection. The projected data in lower dimensions still preserve the information which is held by the original data. The features such as normal and curvature can be obtained by calculating the eigenvalue and eigenvector of covariance matrix in the point set.

Let $P=\left\{p_{1},p_{2},\ldots ,p_{N}\right\}$ be point set with value of X, Y and Z in 3D space, the mean is represented as:

(1) $$\bar{p}=\frac{1}{N}\sum_{i=1}^{N}p_{i}$$

Then, a 3-by-3 covariance matrix can be calculated as:

(2) $$C_{p}=\frac{1}{N}\sum_{i=1}^{N}\left(p_{i}-\bar{p}\right){\left(p_{i}-\bar{p}\right)}^{T}$$

Based on the symmetric property of Equation (2), the covariance matrix can be further rewritten as:

(3) $$C_{p}=M\Lambda M^{T}$$

where M is an orthogonal matrix which is composed of eigenvectors, Λ is a diagonal matrix which is consisted of eigenvalues.

Moreover, Λ can be represented as follows:

(4) $$\Lambda =\left[\begin{array}{ccc} \lambda_{1} & 0 & 0\\ 0 & \lambda_{2} & 0\\ 0 & 0 & \lambda_{3} \end{array}\right]$$

where the components in Equation (4) are eigenvalues satisfying $\lambda_{1}>\lambda_{2}>\lambda_{3}$. 

The eigenvectors of covariance matrix are principal directions that are mutually orthogonal. Besides, the principal curvature which is associated with the first principal component will be applied in the later sections. It can be defined as:

(5) $$\sigma_{1}=\frac{\lambda_{1}}{\lambda_{1}+\lambda_{2}+\lambda_{3}}$$

**Figure 1:**
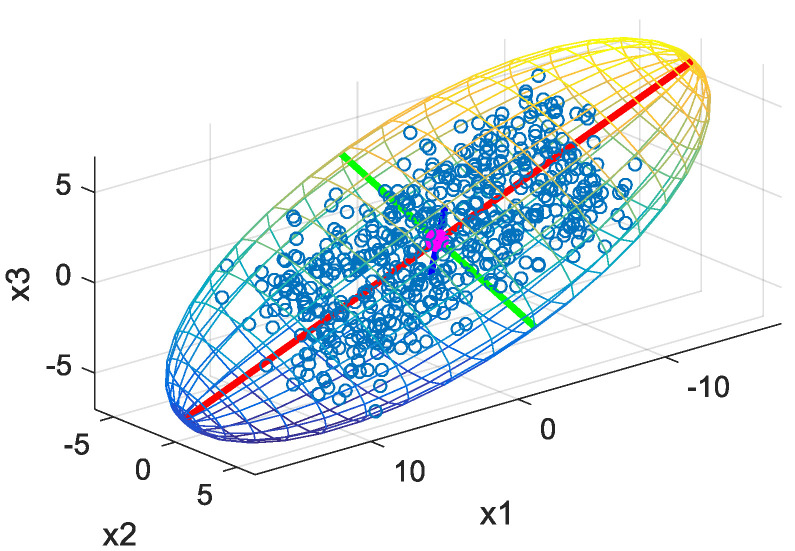
Illustration of principal components in a 3D point set.

Given a 3D point cloud data set, the first principal component corresponds to the orientation of greatest variance as the red line shown in Figure 1. The green and blue lines are associated with second and third principal component, respectively.

### 2.2. k-Nearest Neighbor (kNN)

In this paper, the kNN algorithm [33] is used to search the k-nearest points of every point in a model set to predict the relationship between model set and data set for point cloud registration or in itself to perform PCA. It is a straightforward approach that is simple to implement.

**Figure 2:**
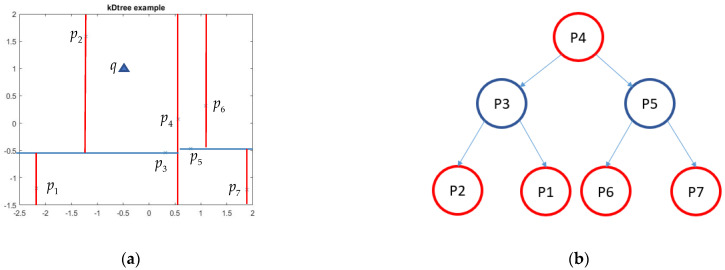
Illustration of kD-tree construction and nearest point search from the kD-tree: (**a**) two-dimensional dataset which has been split based on binary space partitioning; (**b**) representation of a kD-tree as a binary tree.

To achieve the goal of classification by using kNN, a kD-tree [34] needs to be built in advance for the purpose of reducing nearest neighbor searching time. A kD-tree is a binary search tree in k dimensional space which divides the data into subsets. While the process can hardly be interpreted in 3D space. However, the rule of classification and kD-tree construction in 2D space is obvious to realize. Assume that there are seven points $P=\left\{p_{1},p_{2},\ldots ,p_{7}\right\}$ as shown in Figure 2a. The request is inserting a query point q to find its nearest neighbor. According to the rules of kD-tree construction, each node in Figure 2b divides the points with maximum invariance.

Following these rules, the k nearest neighbors, that is, k closest points near a query point can be found. The searching computation time is remarkably reduced due to the characteristic of binary space partitioning. In addition, the kNN algorithm can be implemented rapidly.

### 2.3. Iterative Closest Point (ICP)

In this paper, ICP [34] is the most significant algorithm which is applied for facial point cloud reconstruction and recognition. While performing facial reconstruction, ICP is used to make the surface of point clouds stitched to each other. Besides, while performing face recognition, the angle of view of face point cloud need to be considered. The accuracy may not be desirable without point cloud registration. Therefore, ICP algorithm plays an important role in point cloud preprocessing.

**Figure 3:**
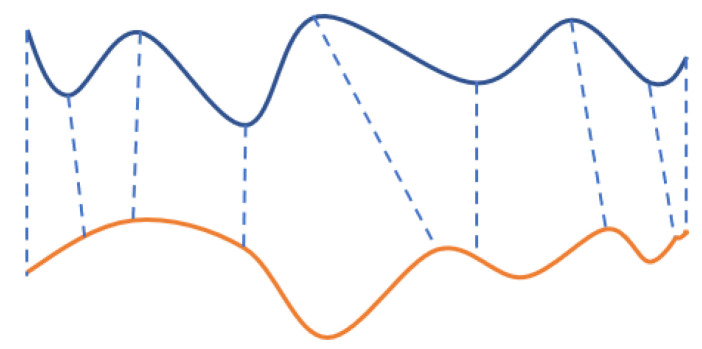
Illustration of 2D point cloud registration.

The aim of ICP algorithm is to best align the point set through spatial transformation, that is, point cloud registration shown in Figure 3. Regarding the point set as rigid body, ICP minimizes the Euclidean distance between the two point clouds through iterations. Precise registration is achieved through ICP. In this study, only the head part point clouds will be considered from the whole frame, since the point clouds are going to be segmented by pre-processing. As a result, the size of the point cloud can be reduced dramatically and therefore the computation loading is affordable.

In 3D space, there are six degrees of freedom, including three-axis translations and three-axis rotations, respectively. The objective function according to the goal of ICP algorithm mentioned above can be defined as:

(6) $$\underset{\left(R,T\right)}{\arg\min E}=\sum_{i=1}^{N}w_{i}{\left\|RP_{i}+T-q_{i}\right\|}^{2}$$

where $P=\left\{p_{1},p_{2},\ldots ,p_{N}\right\}$ is data set and $Q=\left\{q_{1},q_{2},\ldots ,q_{N}\right\}$ is model set. The transformation pair, R and T are rotation matrix and translation vector, respectively. However, outliers may lead to the failure of point cloud registration. Weights for each pair $w_{i}$ is considered in Equation (6) to avoid the effect caused by outliers. It can be defined as:

(7) $$w_{i}=1-\frac{d\left(p_{i},q_{i}\right)}{d_{\max}}$$

where $d\left(p_{i},q_{i}\right)$ is the distance between point pair of nearest neighbor and $d_{\max}$ is maximum distance in the whole point pairs.

Equation (7) illustrates that weight approaches 0 while the distance between point pair is large. Otherwise, it approaches 1. Outliers and inliers can be more clearly distinguished during ICP iterations by applying the concept of weighting.

The centroids of the two point cloud sets are computed as follows:

(8) $$\bar{P}=\frac{\sum_{i=1}^{N}\left(w_{i}p_{i}\right)}{\sum_{i=1}^{N}w_{i}},\;\bar{q}=\frac{\sum_{i=1}^{N}\left(w_{i}q_{i}\right)}{\sum_{i=1}^{N}w_{i}}$$

The optimal translation vector is computed as:

(9) $$T=\bar{q}-R\bar{p}$$

Let ${p^{\prime }}_{i}=p_{i}-\bar{p}$ and ${q^{\prime }}_{i}=q_{i}-\bar{q}$. Taking Equation (9) into Equation (6) and substituting $p_{i}$ and $q_{i}$ into ${p^{\prime }}_{i}$ and ${q^{\prime }}_{i}$, the objective function can be rearranged as:

(10) $$E=\sum_{i=1}^{N}w_{i}{\left\|{p^{\prime }}_{i}\right\|}^{2}-2tr\left(\sum_{i=1}^{N}Rw_{i}{p^{\prime }}_{i}{\left({q^{\prime }}_{i}\right)}^{T}\right)+\sum_{i=1}^{N}w_{i}{\left\|{q^{\prime }}_{i}\right\|}^{2}$$

Let $N=\sum_{i=1}^{N}w_{i}{p^{\prime }}_{i}{\left({q^{\prime }}_{i}\right)}^{T}$, the middle term in Equation (10) become $tr\left(\sum_{i=1}^{N}RN\right)$. Applying the singular value decomposition (SVD) on N yields

(11) $$N=U\sum V^{T}$$

where ∑ is diagonal matrix. The optimal rotation matrix is selected as:

(12) $$R=VU^{T}$$

The optimal transformation pair, T and R is solved based on Equations (9) and (12), respectively.

### 2.4. K-Means Clustering

K-means clustering [23] is an unsupervised learning algorithm which the clusters are based on data without ground truth. The point cloud can be partitioned into parts by using this approach. Given n points and k clusters, then k centroids will be randomly selected. Computing the Euclidean distances between every point and centroid to initially determine every point belongs to the nearest cluster which possess the minimum distance between the centroid. Moreover, the centroids are not fixed. The goal of K-means clustering is to minimize sum of square difference of distance between points and centroids in each group. The objective function can be written as:

(13) $${\underset{\mu }{\arg\min}\sum_{c=1}^{k}\sum_{i=1}^{n}{\left\|p_{i}-\mu_{c}\right\|}^{2}|}_{p_{i}\in S_{c}}$$

where $\mu_{c}$ are group centroids, $p_{i}$ are points in the dataset, and $S_{c}$ are the associated designated groups.

In this paper, a K-means clustering algorithm is applied to segment the upper body point cloud into head and other parts. The detailed processing technique will be introduced in the next section.

### 2.5. Density-Based Spatial Clustering of Applications with Noise (DBSCAN)

DBSCAN [35] is also a clustering algorithm which is based on density. However, different from K-means clustering, DBSCAN doesn’t need to specify quantity of clusters in advance and outliers can be distinguished. In addition, the clusters can be any shape.

**Figure 4:**
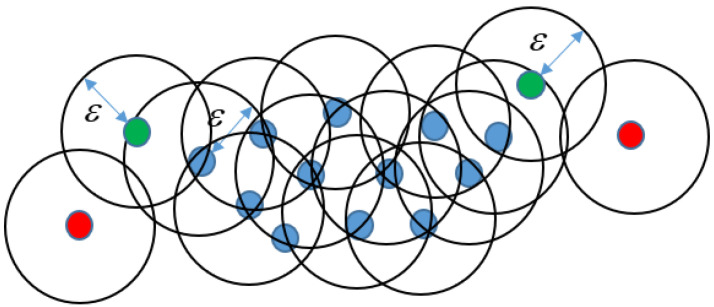
Illustration of density-based spatial clustering of applications with noise (DBSCAN) processing.

There are two parameters that need to be specified, the radius of neighborhood, ε and the minimum number of points, MinPts. The clustering algorithm can be summarized as follows:

1. Expand a circle with radius ε at every point. If the number of points more than MinPts are included in the circle, define the centroid as core point and form a cluster as the blue points shown in Figure 4;
2. If the number of points less than MinPts are included in the circle but another core point is reachable, the centroid will still be involved in a cluster. The centroid is defined as edge point. For example, the green points shown in Figure 4;
3. If there are no points in the circle except for itself as the red points shown in Figure 4. The points are defined as outliers.

In this paper, the DBSCAN is applied to remove outliers. The detailed processing technique will be introduced in the next section.

## 3. 3D Face Point Cloud Reconstruction

In regard to 3D point cloud acquisition, the Microsoft Kinect v2 is set up in our laboratory with indoor and normal light environment. Since any RGB information of the image is not used, the point clouds acquired from depth sensor will not be affected even in dark environments. Lightning is not a problem for data acquisition. The only thing that one needs to be careful about is that the depth sensor should not be irradiated by sun illumination directly because the sensor may not work, and a large part of point cloud will be lost.

**Figure 5:**
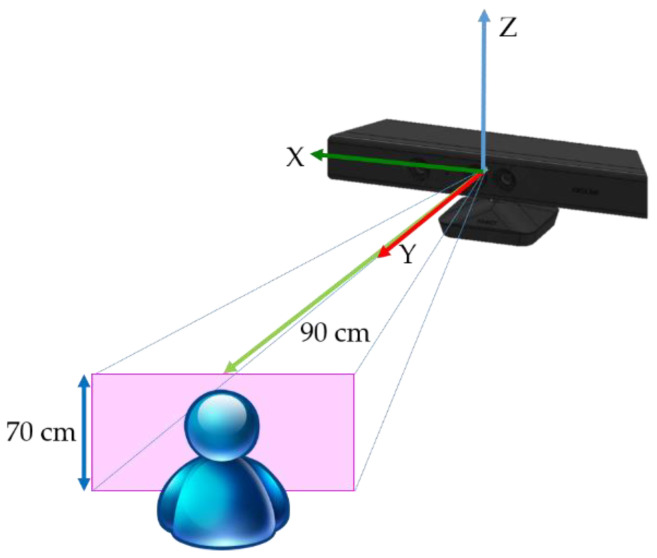
The configuration of data set acquisition using Microsoft Kinect v2.

The illustration of the initial configuration and setup of Microsoft Kinect v2 is shown in Figure 5. The distance between model and sensor is approximately 90 cm and the associated density of point cloud is optimal via test. It is recommended that the average distance between points in the point cloud should not exceed 2 mm. Most of the texture such as the eyes, nose and mouth can be clearly revealed. If the model is too close to the sensor, due to the limit of the field of view (FOV) of the depth sensor, the face point may not be collected properly. On the contrary, if the model is placed too far from the depth sensor, sparse point cloud will be obtained, in which the point cloud may not describes the face detail texture clearly.

**Figure 6:**
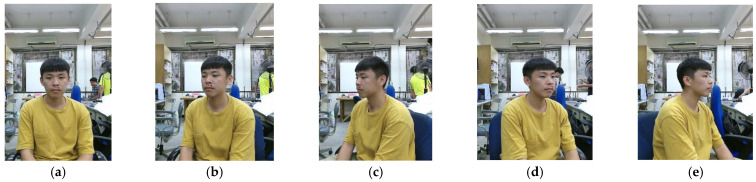
2D RGB images of different angle of rotation relative to camera: (**a**) front view (A); (**b**) turn left 30 degrees (B); (**c**) turn left 60 degrees (C); (**d**) turn right 30 degrees (D); (**e**) turn right 60 degrees (E).

For all the test members taken as the data base, multi-view face point clouds are going to reconstruct a denser model set first. For a single test member, there are five total point clouds needed; the front view, turn left 30 degrees, turn left 60 degrees, turn right 30 degrees and turn right 60 degrees relative to the depth camera, respectively. An example illustration of the model data set collection is shown in Figure 6. To simplify naming confusion of point cloud, let A be the point cloud corresponding to Figure 6a, B be the point cloud corresponding to Figure 6b, and so on. These abbreviations will be used in Section 3.2.

The reconstruction technique can be divided into three parts; head segmentation, point cloud registration and denoising.

### 3.1. Head Segmentation

**Figure 7:**
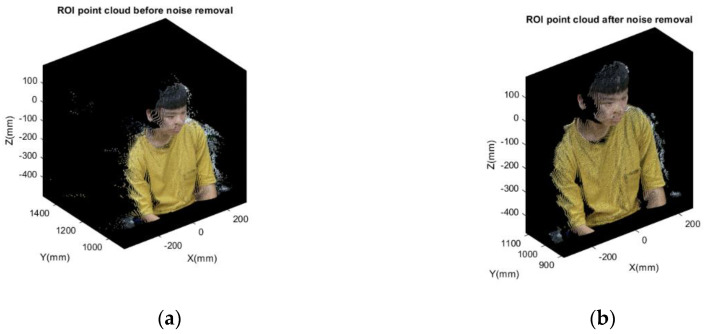
The region of interest (ROI) point cloud before and after noise removal: (**a**) before noise removal; (**b**) after noise removal.

Since the point cloud not only contains face model we need, certain background objects within the range of 5 m [36] in front of depth camera are also included. Thus, establishing a region of interest (ROI) is necessary to focus on the model processing and to remove meaningless background objects. According to the initial configuration shown in Figure 5, preliminary filtering can be applied by using the intrinsic function in MATLAB based on [37], the function “pcdenoise”. The function is applied for the point cloud noise removal after initially acquiring the data set. There are two parameters needing to be specified; the number of nearest neighbor points and the outlier threshold. The first one evaluates the mean of average distance between all points and specified number of nearest points. The second parameter specifies the standard deviation from mean average distance to neighbors of all points. Once the mean distance from a query point to its neighbors is larger than the threshold, it is considered an outlier. Removing the outliers and a lower-noise point cloud can be acquired, which is enough for the following point cloud processing such as 3D face reconstruction and recognition. The preliminary filtering result is shown below. Figure 7 shows the ROI point cloud before and after noise removal. It is obvious that most of the sparse outliers can be removed.

Because the dimension of the head may be different for every test member, it is hard to set a fixed ROI, which may include unnecessary parts such as the neck, arm and chest, when applying the head segmentation directly. Therefore, a further precise head segmentation is needed. 

**Figure 8:**
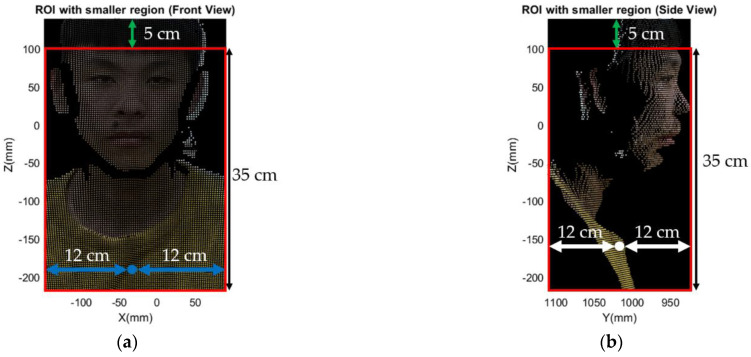
Rough segmentation of ROI point cloud: (**a**) extend 12 cm back and forth from mean of X axis; (**b**) extend 12 cm back and forth from mean of Y axis.

First, in order to achieve the precise head segmentation, refining a ROI which confines a smaller region is needed. Note that the ROI has a longer length in the Z axis and symmetry in the X axis and Y axis to prepare for precise segmentation through the K-means algorithm later. Regarding the determination of the ROI, the mean values of X and Y coordinate in ROI point cloud is calculated. Next, extending 12 cm back-and-forth and left-and-right with respect to the mean, as the blue and white lines shown in Figure 8a,b, respectively. After that, the region of 5 cm from top of the point cloud is excluded to preserve the face without hair. Finally, the region with 35 cm height from the forehead is preserved. Figure 8 gives the complete illustration of rough segmentation as mentioned above. Later, K-means clustering is applied for further precise head segmentation.

**Figure 9:**
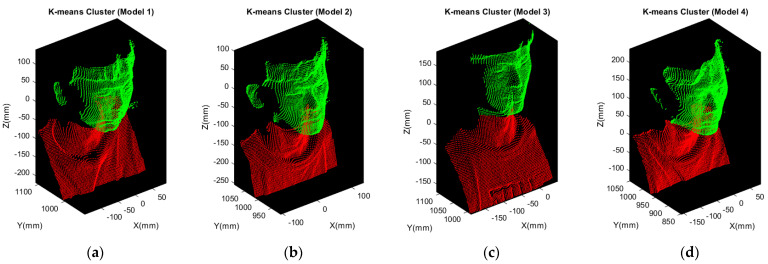
Precise segmentation by K-means clustering: (**a**) head point cloud of Model 1; (**b**) head point cloud of Model 2; (**c**) head point cloud of Model 3; (**d**) head point cloud of Model 4.

Based on the rough segmentation completed, K-means clustering is ready for further precise head segmentation. Referring to Figure 8a,b, it evidently shows that there is an obvious gap between the chin and neck. Besides, according to the shape, narrow region along X and Y direction and longer length along Z direction bounded by the red bounding box in Figure 8, which means that head can be simply segmented with two clusters implemented by K-means clustering. Figure 9 demonstrates the results of precise head segmentation and verifies that the presented segmentation algorithm described in Section 3.1 is suitable even for different people. Head can be simply segmented with setting two clusters for K-means clustering because it is applicable to the human body contour. The point cloud marked in red and green is the result of implementing K-means clustering.

Next, the clusters marked in red are going to be discarded and then the complete head point clouds can be extracted accordingly. These four models are then further used for 3D face reconstruction as presented in Section 3.2. 

### 3.2. Point Cloud Registration

To integrate the segmented point cloud into a complete face point cloud, the ICP algorithm is used for multi-view point clouds registration. Note that the given two point cloud sets without initial alignment may diverge during iteration because the distance between two point cloud sets is too far, which leads to the failure of nearest neighbor searching. The alignment technique will be introduced later. 

Therefore, we detect the nose tip of A, B, D point cloud in Figure 6 by using shape index with curvature. According to [38], shape index is used to determine the shape of a 3D object. The shape index at a point p can be defined as:

(14) $$S_{I}\left(p\right)=\frac{1}{2}-\frac{1}{\pi }\tan^{-1}\frac{\sigma_{1}\left(p\right)+\sigma_{3}\left(p\right)}{\sigma_{1}\left(p\right)-\sigma_{3}\left(p\right)}$$

where $\sigma_{1}\left(p\right)$ is the principal curvature as shown in Equation (5) and $\sigma_{3}\left(p\right)$ is the third curvature which is associated with the third principal component, it can be written as:

(15) $$\sigma_{3}=\frac{\lambda_{3}}{\lambda_{1}+\lambda_{2}+\lambda_{3}}$$

**Figure 10:**
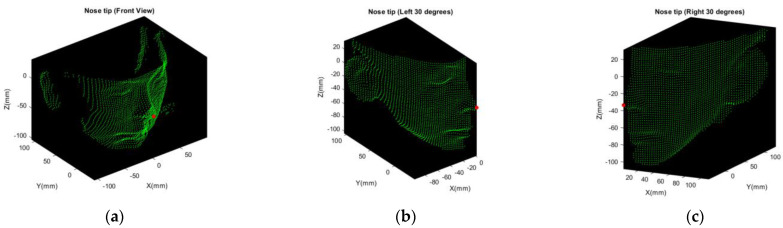
Multi-view nose tip detection, all nose tip is marked as red point: (**a**) nose tip of A point cloud (front view); (**b**) nose tip of B point cloud (turn left 30 degrees); (**c**) nose tip of D point cloud (turn right 30 degrees).

Based on the definition of shape index in Equation (14), it can represent the local shape of an object continuously between concave and convex. The shape index is larger while the local shape is similar to a dome or spherical cap. Therefore, nose tip is the point with the larger value of shape index, shown as the red points shown in Figure 10. 

**Figure 11:**
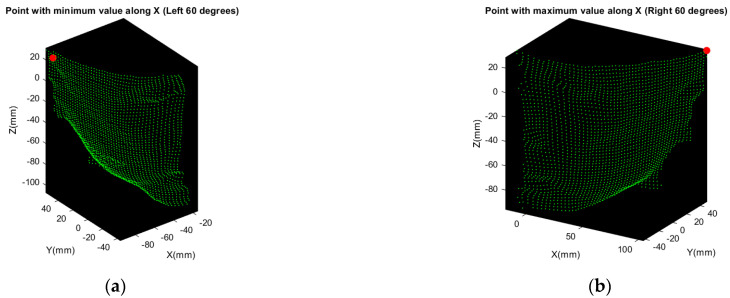
Detection of extremum points along X direction, both are marked as red point: (**a**) point with minimum value along X axis of C point cloud; (**b**) point with maximum value along X axis of E point cloud.

The angle of rotation of C and E point cloud in Figure 6 is larger so that the nose tip may not be involved. However, we detect the point with maximum or minimum value along X direction, as the red points shown in Figure 11.

**Figure 12:**
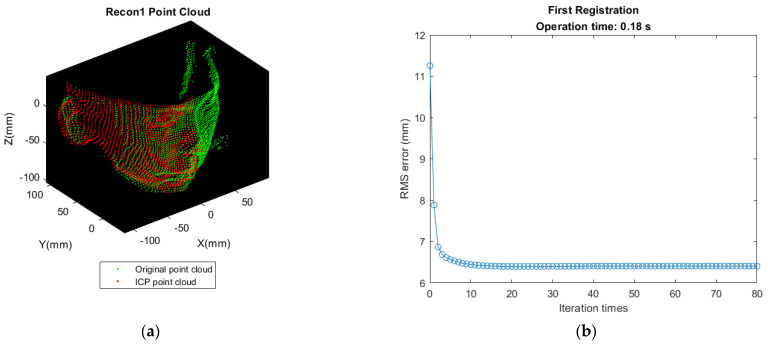
Result of first registration and deviation. (**a**) Recon 1 Cloud shows that some losing information is compensated by B point cloud; (**b**) ICP iteration is converged about ten times.

**Figure 13:**
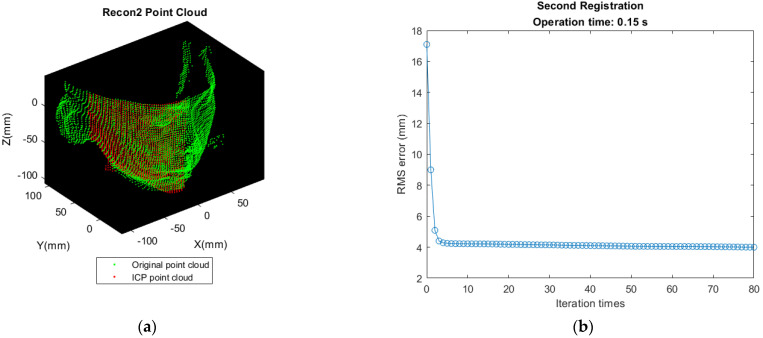
Result of second registration and deviation. (**a**) Recon 2 Cloud shows that partial features at left face have been reinforced after matching; (**b**) ICP iteration is converged about ten times.

**Figure 14:**
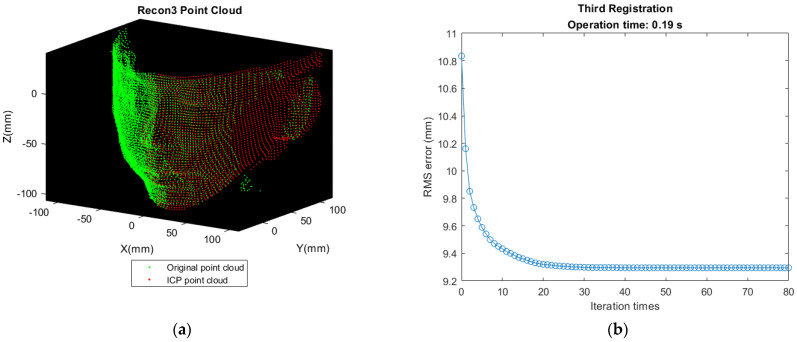
Result of third registration and deviation. (**a**) Recon 3 Cloud shows that some losing information is compensated by D point cloud. (**b**) The RMS error is larger because of sparser distribution of points and more outliers on the right face.

**Figure 15:**
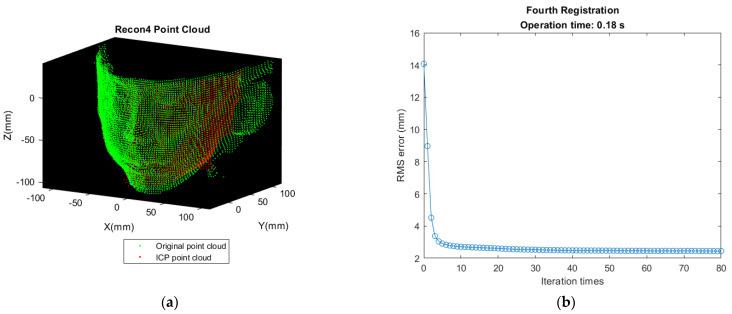
Result of fourth registration and deviation. (**a**) Recon 4 Cloud shows that partial features at right face have been reinforced. (**b**) The RMS error becomes small because of denser Recon 3 Cloud.

After reference points for initial alignment are found, four times ICP registration is to be performed. The alignment techniques and matching steps are summarized as:

1. First matching the front-view point cloud (A point cloud) with B point cloud in Figure 6b. Transforming B’s nose tip to the same position as A’s nose tip for initial alignment. Let it be Recon 1 Cloud after performing ICP. The result of registration and deviation are shown as Figure 12.
2. Detecting the point with minimum value along X direction in Recon 1 Cloud and translating the point found in Figure 11a to the same position as this point for initial alignment. Let it be Recon 2 Cloud after performing ICP. The result of registration and deviation are shown in Figure 13.
3. So far, the reconstruction of the left half of the face is completed. The reconstruction approach is same for the right half of the face. Detecting the nose tip in Recon 2 Cloud, then translating D’s nose tip to the location same as this point for initial alignment. Let it be Recon 3 Cloud after performing ICP. The result of registration and deviation are shown in Figure 14. However, the deviation is larger as shown in Figure 14b, which may be affected by the sparser distribution of points and more outliers in the right face.
4. Translating the point found in Figure 11b to the same position as the point with the maximum value along X direction in Recon 3 Cloud for initial alignment. Then, let it be the Recon 4 Cloud after performing ICP. The result of registration and deviation are shown in Figure 15.

The techniques of 3D facial point cloud reconstruction have been illustrated above in detail. However, the growing outliers may lead to misclassification while performing face recognition. As a consequence, point cloud denoising is necessary to remove the outliers. Related processing will be introduced in Section 3.3.

### 3.3. Point Cloud Denoising

**Figure 16:**
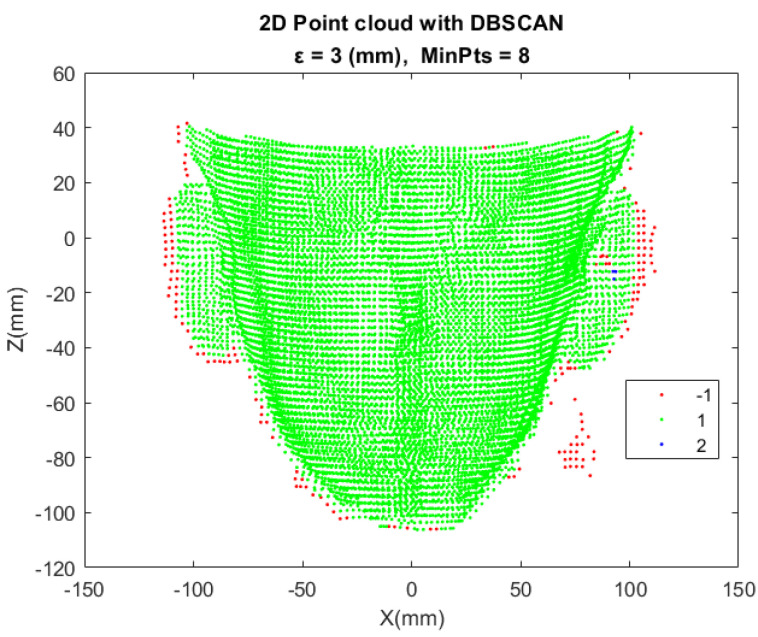
The 2D point cloud after DBSCAN processing.

**Figure 17:**
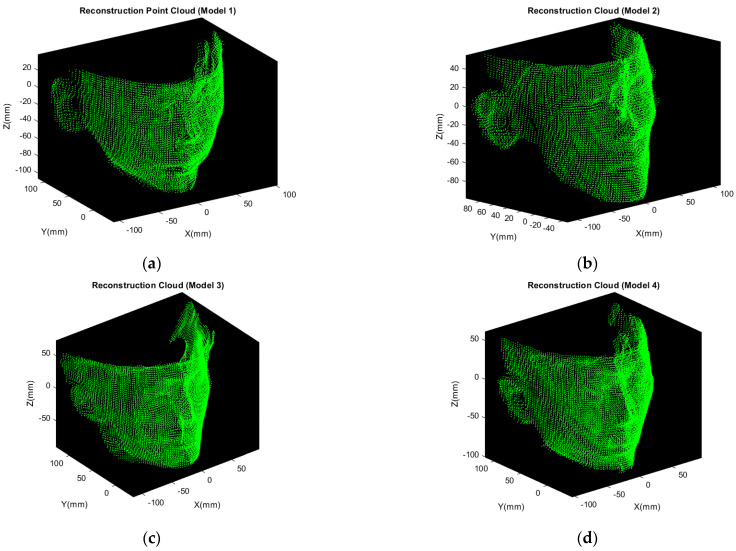
3D face reconstruction of four laboratory members: (**a**) Model 1; (**b**) Model 2; (**c**) Model 3; (**d**) Model 4.

Since the reconstructed model contains many outliers as shown in Figure 15a, the aforementioned DBSCAN is used for noise removal. Regarding outlier removal, the trick is, only the X-Z plane information is adopted. It is the projection of the Recon 4 Cloud onto the X-Z plane. Apply DBSCAN algorithm to this 2D point cloud. The result is shown in Figure 16, where the red point clouds are going to be discarded and the green ones are going to be preserved. By applying the same procedure, the final face point cloud reconstruction of all the models are shown in Figure 17.

The models are reconstructed from five multi-view point clouds, namely, the models shown in Figure 17 in this paper are still point clouds but not mesh. To statistically analyze the point clouds before and after reconstruction, the nearest neighbor search is applied to compute average distance of the point clouds. Taking the average of sum of the distance between each point and its nearest neighbor, average distance can be acquired. It can be represented as:

(16) $$Average\;dist.=\frac{\sum_{i=1}^{N}\min\;dist.\left(p_{i},q_{i}\right)}{N}$$

where N is the number of points and $\min dist.\left(p_{i},q_{i}\right)$ is the minimum distance between point pair of nearest neighbor.

The comparison between original point cloud and reconstruction model is tabulated in Table 1. It shows that the average distance between each point and its nearest neighbor is lower between and after reconstruction. The denser the point cloud, the more features can provide. The textures can also be present clearly with dense reconstruction model.

**Figure 18:**
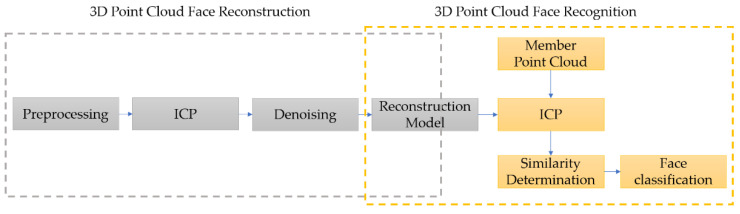
Flowchart of 3D facial reconstruction and recognition.

These reconstruction models displayed in Figure 17 will be taken as the model set in database. The complete flowchart of face reconstruction and recognition is summarized as in Figure 18.

**Figure 19:**
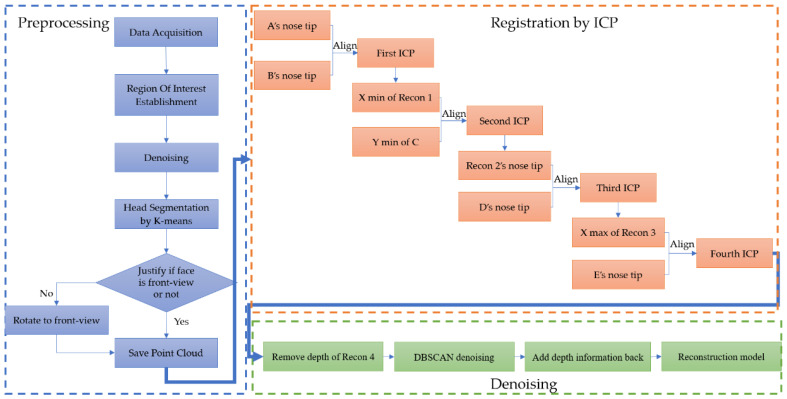
Flowchart of 3D point cloud facial reconstruction, the blue block diagram shows the preprocessing steps, the orange block diagram shows the registration steps and the green block diagram shows the denoising procedures.

Among the part of 3D point cloud facial reconstruction, the preprocessing steps from the very beginning can be summarized as the blue block diagram at the left block shown in Figure 19. The registration steps are shown in the orange block diagram on the upper right block of Figure 19. The reconstruction point cloud denoising steps are shown in the green block diagram at the lower right part of Figure 19.

The reconstruction point clouds will be taken as models in database for face recognition. Calculating the similarity score of the data set from current shot and comparing it with each model set in the database will be the next milestone. The most similar candidates can be chosen after comparing the scores. The similarity score and criterion for 3D face recognition will be introduced in the next section.

## 4. Face Score for Classification

### 4.1. Point Cloud Similarity

In this section, the 3D facial point cloud recognition is going to be performed after the model set reconstruction is completed. The principal curvature, normal vector computed, and registration error are applied to determine the similarity of different facial point clouds. The advantage of using this method is that facial recognition can still be performed even the data set from current shot may have tilted, namely, it’s invariant to rotation.

#### 4.1.1. Normal Similarity Score

The continuous distribution of normal can describe the shape of an object. Therefore, it is feasible to adopt the normal as a benchmark to compare the surface normal difference between two given point cloud sets. The formula of normal similarity score in [39] is applied in this paper. 

Assume that point cloud registration has been done by the ICP. The normal of every point in the two point clouds itself will then be calculated. Let the model point cloud be $Q=\left\{q_{1},q_{2},\ldots ,q_{n}\right\}$ and the data point cloud from current shot be $P=\left\{p_{1},p_{2},\ldots ,p_{n}\right\}$. The normal of every point, which can be calculated through PCA, is stated as:

(17) $$f\left(x\right)=\vec{n}\left(x\right)$$

The nearest point pair searching by kNN algorithm between two point cloud sets is represented as:

(18) $$r=\left\{\left(q_{1},p_{1}\right),\left(q_{2},p_{2}\right),\ldots ,\left(q_{n},p_{n}\right)\right\}$$

According to [39], the normal similarity is defined as:

(19) $$\begin{array}{ll} s_{normal}\left(r,f\right) & =s_{normal}\left(q,p,f\right)\\ & =\frac{\left\{\pi -\cos^{-1}\left[f\left(q\right)\cdot f\left(p\right)\right]\right\}}{\pi }\\ & =\frac{\left\{\pi -\cos^{-1}\left[\vec{n}\left(q\right)\cdot \vec{n}\left(p\right)\right]\right\}}{\pi } \end{array}$$

From Equation (19), the normal similarity is largest when $s_{normal}$ has the maximum value, 1, which means that the included angle between two normal is zero; however, $s_{normal}=0$ when the angle between two normal is 180 degrees.

In addition, the direction of normal vector should also be careful. To ensure the consistency of direction with each other, the normal vector need to be flipped if the angle between two vectors is larger than 90. 

#### 4.1.2. Curvature Similarity Score

The normal represents the orientation of the plane formed by points in the local region; however, the curvature represents the convexity or concavity of surface, namely, it describes the rate of change in tangent plane. The same object has a similar curvature and normal. Therefore, the shape of a point cloud can be completely presented if the curvature as well as normal are both taken into consideration for similarity evaluation.

According to the eigenvalues, $\lambda_{1}$, $\lambda_{2}$ and $\lambda_{3}$ which calculated by PCA in Section 2.1, the principal curvature can be defined as:

(20) $$g\left(\lambda \right)=\frac{\lambda_{1}}{\lambda_{1}+\lambda_{2}+\lambda_{3}}$$

where $\lambda_{1}>\lambda_{2}>\lambda_{3}$ and 0<g(λ)<1.

Using the same approach and symbols as in Section 4.1.1, search the nearest point pair r after the model point cloud, Q, and the data point cloud from current shot, P, have been matched through ICP. According to [39], the curvature similarity, $s_{curvature}$ is defined as:

(21) $$\begin{array}{ll} s_{curvature}\left(r,g\right) & =s_{curvature}\left(q,p,g\right)\\ & =1-\left|g\left(q\right)-g\left(p\right)\right| \end{array}$$

From Equation (21), $s_{curvature}$ approaches 1 when curvature of the pair is similar. Otherwise, $s_{curvature}$ approaches 0.

#### 4.1.3. Registration Similarity Score

In this paper, we propose a novel approach to determine the similarity of point clouds before and after registration. The sum of the Euclidean distance difference of every point will be acquired in each ICP iteration, namely, objective function E in Equation (6). The value of E generally descends during iterations, which means the two point clouds get closer. The registration similarity is defined as:

(22) $$s_{icp}=1-\frac{E_{final}}{E_{initial}}$$

where $E_{initial}$ is generally the maximum distance difference of model set and point set before registration. $E_{final}$ is the minimum distance difference obtained from the last ICP iteration.

Equation (22) illustrates how close the given two point cloud sets are after registration. $E_{initial}$ becomes large if the data set may initially have large angle of rotation or translation relative to model set. Moreover, $E_{final}$ becomes small whether the data and model set is same person. Hence, $s_{icp}$ approaches 1 when the two point clouds are getting closer after registration.

### 4.2. Face Similarity Score

In this paper, the face similarity score is composed of normal, curvature and registration similarity as mentioned above. Let the model set be Q and data set be P, define the composite face similarity score as follows

(23) $$S_{face}\left(q,p\right)=100\times \left[w_{1}\times \frac{\sum_{i=1}^{n}s_{normal}\left(q_{i},p_{i},f\right)}{n}+w_{2}\times \frac{\sum_{i=1}^{n}s_{curvature}\left(q_{i},p_{i},g\right)}{n}+w_{3}\times s_{icp}\right]$$

where n is the number of point pairs, $w_{1}$, $w_{2}$ and $w_{3}$ are weights for different similarity determination. The value of the weights is between 0 and 1. Besides, the sum of weights is 1.

According to different dataset, the value of weights may be different. The detailed justification of weights and criterion for face recognition will be introduced in the next section.

## 5. Experiment Verification

**Figure 20:**
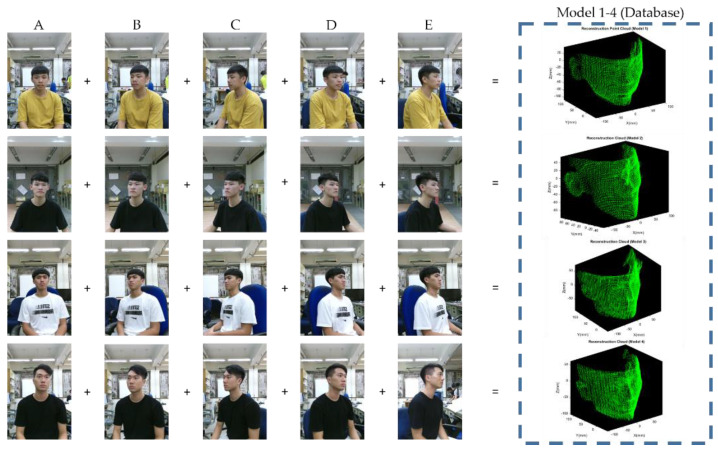
Schematic illustration of model point cloud reconstruction.

The five frames {A, B, C, D, E} in Section 3 are acquired in multi-view geometry, which is applied for model point cloud reconstruction only. The reconstruction point cloud in Figure 17 will be taken as model 1–4 in database. The schematic illustration is shown in Figure 20.

**Figure 21:**
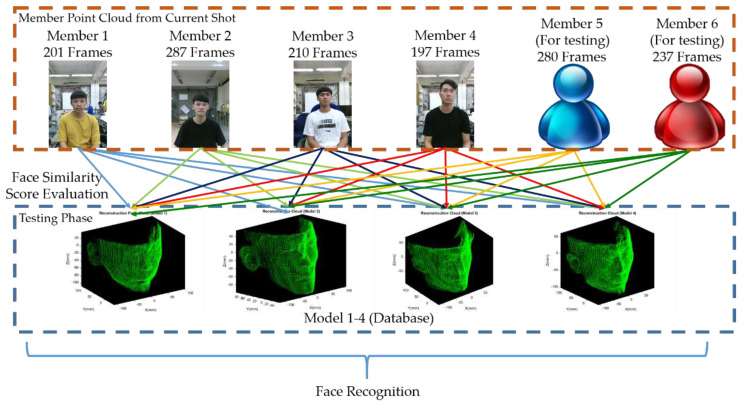
Schematic illustration of 3D face recognition.

However, this section illustrates the experimental verification to test the feasibility of 3D face recognition, which is applied for some specific fields such as our laboratory and office for security purpose. To simplify naming confusion, models in database are named as Model 1–4 and the testing point clouds for 3D face recognition are named as Member 1–6. The schematic illustration is shown in Figure 21.

**Figure 22:**
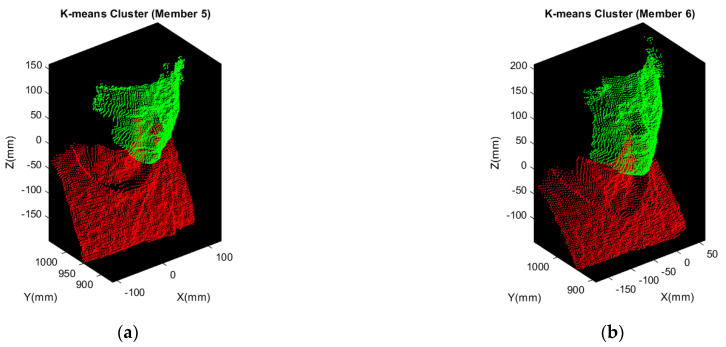
Illustration of Member 5 and Member 6. (**a**) Head point cloud of Member 5; (**b**) head point cloud of Member 6.

To test the feasibility of recognition using similarity score, we acquired plenty of data sets to perform 3D facial recognition. Among the Member point cloud, Member 1–4 correspond to the same person as Model 1–4. In the testing phase, each frame of the Member point cloud will be registered with Models 1–4 and a similarity score with every Model is evaluated. In addition, to test whether the member not in database can still be classified or not, there are Member 5 and Member 6 not in database as shown in Figure 22, which have 280 and 237 frames, respectively. Each frame of Members 5–6 will also be registered with Models 1–4 and similarity score with every Model is evaluated.

The quantity of data set is tabulated in Table 2 so that it’s easier to understand. There are total of 1412 frames in data set to test the feasibility of 3D face recognition using similarity score.

**Figure 23:**
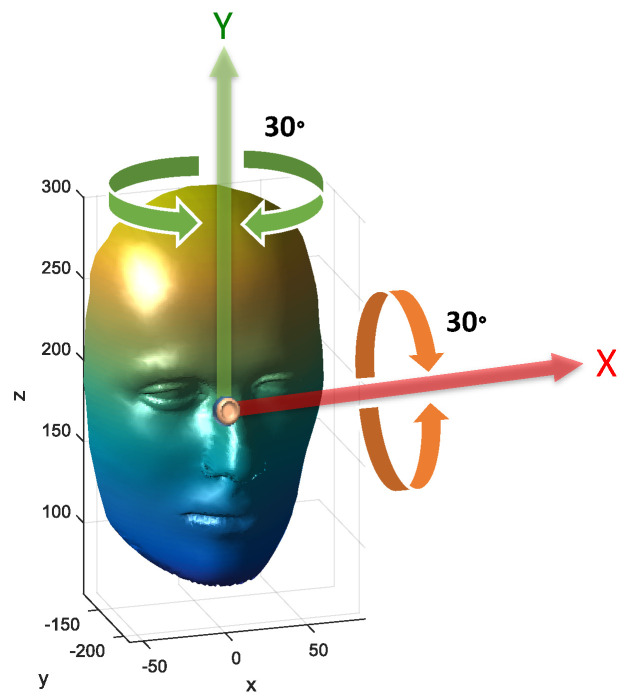
Illustration of 3D facial point cloud pose.

To simulate the situation of 3D facial recognition on smart devices and to challenge whether the 3D point cloud face recognition are robust against pose and rotation or not, the rotation range $\pm 30^{{}^{\circ}}$ in roll/pitch angles is considered, which is illustrated in Figure 23. Indeed, the facial similarity score will be the highest if the face is facing the sensor without obvious rotations. However, the presented 3D facial recognition can still work properly with $\pm 30^{{}^{\circ}}$ in roll/pitch angles owing to the feature aligned ICP registration. Related experiments demonstrate that even in the presence of facial rotations, the developed face recognition algorithm can also provide good accuracy.

Taking each data set as input, calculating the face similarity score mentioned in Section 4.2 with every model set in database. The criteria for facial point cloud recognition are listed as follows:

1. Identify as the same person if face similarity score with data set from current shot and one of model set in database is greater than 80, namely, $S_{face}(q,p)>80$;
2. If a data set from the current shot has face similarity score larger 80 with more than two model set, identifying the model with highest score, which means the most similar model in the database;
3. Otherwise, none of the models will be identified if the data set from the current shot has a face similarity score less than 80 with every model set.

The criteria above are inductions according to our experiment. In the experiment, we found that curvature is the most important parameter which reveals the difference between the data set and model set. In addition, the recognition similarity score helps us widen the difference in highest score and second highest score. Thus, we define the values of each parameter in similarity score are:

(24) $$w_{1}=0.15,\;w_{2}=0.6,\;w_{3}=0.25$$

**Figure 24:**
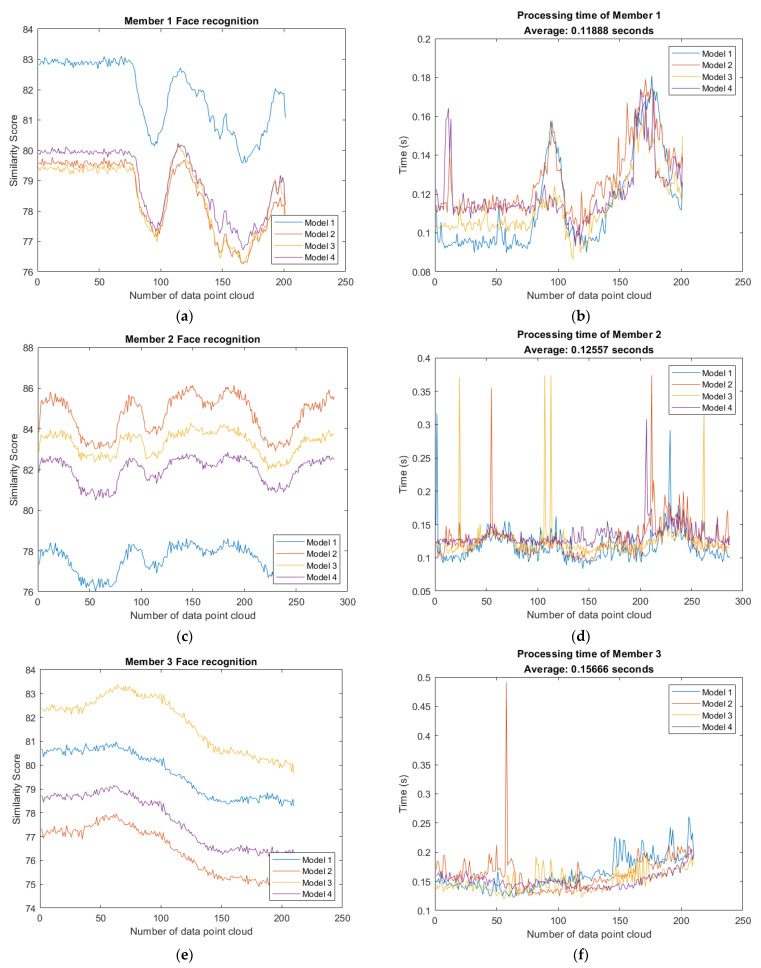

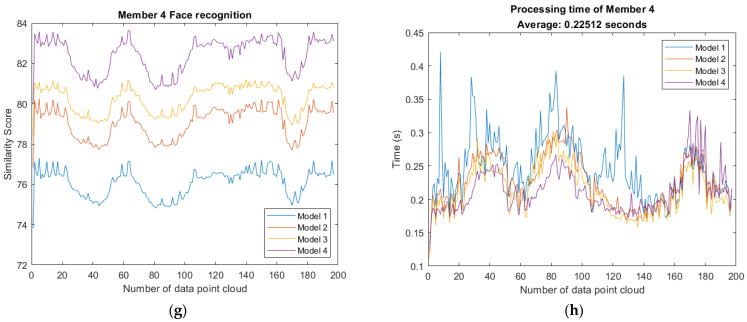
Result of 3D face recognition: (**a**) 12 frames of Member 1 are recognized as none; (**b**) computation time of each frame with Member 1; (**c**) every Member 2 data set is recognized as Model 2; (**d**) computation time of each frame with Member 2; (**e**) 19 frames of Member 3 are recognized as none; (**f**) computation time of each frame with Member 3; (**g**) only one frame of Member 4 is misclassified as none; (**h**) computation time of each frame with Member 4.

The result of 3D face recognition and processing time is shown in Figure 24. Figure 24a–d shows the accuracy between Member 1 to Member 4 and every model set. There are 12 frames of Member 1 recognized as none, which the face similarity score is less than 80. The accuracy is 94.03%. Every data set of Member 2 is recognized as Model 2, the accuracy is 100%. In Figure 24c, there are 19 frames of Member 3 recognized as none, the accuracy is 90.95%. Besides, only one frame of Member 4 is misclassified as none, the accuracy is 99.49%. In addition, the average processing time is also computed and displayed on the title. The similarity score can be evaluated within less than 0.23 s with the algorithm presented in this paper. 

**Figure 25:**
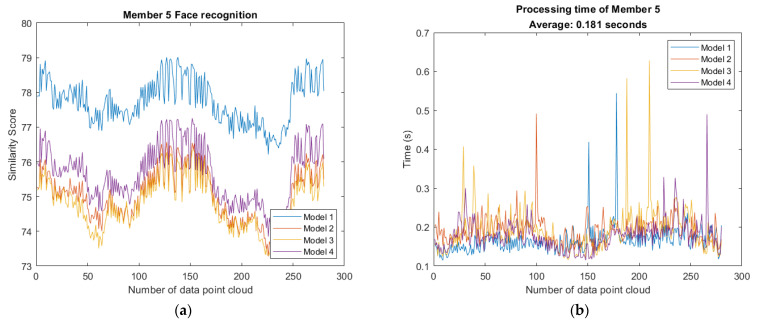

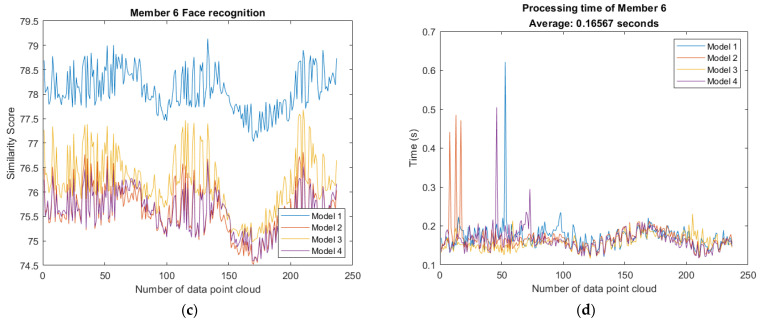
Result of 3D face recognition: (**a**) none of Model is recognized as Member 5; (**b**) computation time of each frame with Member 5; (**c**) none of Model is recognized as Member 6; (**d**) computation time of each frame with Member 6.

The result of 3D face recognition with members not in database is shown in Figure 25. Figure 25c,d show the experiment with the member not in the database. It is evident that none of Member 5 and Member 6 is classified as model because none of the face similarity score of data set is greater than 80. Members not in the database are not misclassified and thereby the examination accuracy is 100%. 

According to the result of 3D face recognition and processing time as shown in Figure 24 and Figure 25, it proves that the algorithm is competitive with fast computation and high accuracy. Table 3 summarize the relationship between model, frames of member and accuracy.

## 6. Conclusions

This work presents a computation efficient point cloud preprocessing procedure for facial point cloud reconstruction and recognition. The main motive is to solve the facial recognition problem in light unstable or dark environments. Moreover, the computation is efficient and therefore it is suitable for implementation in smart devices with depth cameras. In the preprocessing phase, the technique of ROI confinement that preparing for K-means clustering is introduced. Precise head segmentation can be achieved by applying this approach. In the registration phase, multi-view point clouds are used to rebuild high-density face point clouds using the ICP, in which initial alignment skills are also presented. However, the existence of outliers may degrade the performance of recognition and therefore denoising is necessary to apply. In the denoising phase, a simple method is proposed to detect noise. Projecting the point cloud in the XZ plane and implementing the DBSCAN algorithm, the outliers can be simply detected. Taking the reconstruction point cloud as the model set and comparing the similarity between the data set from current shot and model set. A robust and simple method, a facial similarity score with a novel registration similarity score are presented to classify the face point cloud. For the test data sets which are not included in the training database, they will not be misclassified. Experiment illustrates the robustness of the presented approach. Briefly, in this paper an effective procedure for 3D facial reconstruction/recognition is proposed with fast computation and high accuracy. Experiments with real world data shows the feasibility of our method. All algorithms are implemented without using high cost deep learning skills or any RGB information. Complicated training is not needed to improve the accuracy, but almost 100% accuracy can be still achieved. The proposed method can not only decrease the computational time and complexity significantly, but also can be applied in light insufficient or even dark environments.

## Figures and Tables

**Table 1:** Comparison between the original point cloud and reconstruction model.

Model	Avg. Distance of Original Point Cloud (mm)	Avg. Number of Points in Original Point Cloud	Avg. Distance of Reconstruction Model (mm)	Number of Points in Reconstruction Model
Model 1	2.8361	2509	1.0874	12,381
Model 2	2.8237	3193	0.9623	14,882
Model 3	2.9083	3175	1.1992	15,813
Model 4	2.6547	2741	0.9811	13,602

**Table 2:** Frames of each member in data set.

Member	Frames
Member 1 (Same person as model 1)	201
Member 2 (Same person as model 2)	287
Member 3 (Same person as model 3)	210
Member 4 (Same person as model 4)	197
Member 5 (Not in database for testing)	280
Member 6 (Not in database for testing)	237

**Table 3:** Accuracy of 3D face recognition.

Member	Model 1	Model 2	Model 3	Model 4	None	Accuracy
Member 1	189	0	0	0	12	94.03%
Member 2	0	287	0	0	0	100%
Member 3	0	0	191	0	19	90.95%
Member 4	0	0	0	196	1	99.49%
Member 5	0	0	0	0	280	100%
Member 6	0	0	0	0	237	100%

## Data Availability

Non-digital data available.
